# Fine mapping and functional validation of the maize nicosulfuron-resistance gene *CYP81A9*

**DOI:** 10.3389/fpls.2024.1443413

**Published:** 2024-08-01

**Authors:** Yongzhong Zhang, Qingrong Zhang, Qingzhi Liu, Yan Zhao, Wei Xu, Cuiping Hong, Changli Xu, Xiushan Qi, Xinli Qi, Baoshen Liu

**Affiliations:** ^1^ College of Agronomy, Shandong Agricultural University, Taian, Shandong, China; ^2^ Qingdao Academy of Agricultural Sciences, Qingdao, Shandong, China; ^3^ Department of Maize Breeding, Taian Denghai WuYue Taishan Seed Industry CO., LTD, Taian, Shandong, China

**Keywords:** maize, nicosulfuron resistance, P450, *CYP81A9*, ALS

## Abstract

Nicosulfuron, a widely utilized herbicide, is detrimental to some maize varieties due to their sensitivity. Developing tolerant varieties with resistance genes is an economical and effective way to alleviate phytotoxicity. In this study, map-based cloning revealed that the maize resistance gene to nicosulfuron is *Zm00001eb214410* (*CYP81A9*), which encodes a cytochrome P450 monooxygenase. qRT- PCR results showed that *CYP81A9* expression in the susceptible line JS188 was significantly reduced compared to the resistant line B73 during 0-192 hours following 80 mg/L nicosulfuron spraying. Meanwhile, a *CYP81A9* overexpression line exhibited normal growth under a 20-fold nicosulfuron concentration (1600 mg/L), while the transgenic acceptor background material Zong31 did not survive. Correspondingly, silencing *CYP81A9* through CRISPR/Cas9 mutagenesis and premature transcription termination mutant EMS4-06e182 resulted in the loss of nicosulfuron resistance in maize. Acetolactate Synthase (ALS), the target enzyme of nicosulfuron, exhibited significantly reduced activity in the roots, stems, and leaves of susceptible maize post-nicosulfuron spraying. The *CYP81A9* expression in the susceptible material was positively correlated with ALS activity *in vivo*. Therefore, this study identified *CYP81A9* as the key gene regulating nicosulfuron resistance in maize and discovered three distinct haplotypes of *CYP81A9*, thereby laying a solid foundation for further exploration of the underlying resistance mechanisms.

## Introduction

Maize, a globally essential food and feed crop, encounters significant production challenges due to weed infestation. The primary method for controlling these weeds is the application of herbicides (Ferrero et al., 2017). Among the various herbicides, the sulfonylurea herbicide nicosulfuron is highly effective against Poaceae grasses. Nicosulfuron is one of the least damaging herbicides for maize varieties, typically applied at the seedling stage to effectively control weeds (Kurniadie et al., 2023).

Nicosulfuron is a systemic herbicide that targets acetolactate synthase (ALS) through both xylem and phloem transport, effectively inhibiting an essential metabolic pathway and disrupting weed growth. By inhibiting ALS activity, nicosulfuron prevents pyruvate condensation reactions, thereby significantly reducing the synthesis of branched-chain amino acids such as valine, leucine, and isoleucine. This disruption of essential metabolic processes inhibits weed growth (Tan et al., 2006; Chakraborty et al., 2020). Ultimately causing the withering and eventual death of weeds and susceptible maize varieties. In contrast, resistant maize varieties exhibit enhanced photosynthetic capabilities and reactive oxygen species scavenging capacities, allowing them to withstand the nicosulfuron-induced effects. Additionally, resistant maize rapidly degrades nicosulfuron into inactive substances, thereby mitigating its harmful effects (Wang et al., 2021; Wu et al., 2022).

Nicosulfuron metabolism via hydroxylation is similar to the enzymatic activity of cytochrome P450 (CYP450) in tolerant maize plants (Kreuz et al., 1996). CYP450s are prevalent in animal and plant cells, where they are primarily associated with membrane-bound organelles such as the endoplasmic reticulum, mitochondria, plastids, and Golgi apparatus (Lu et al., 2015). As terminal oxygenase, CYP450 enzymes play a pivotal role in numerous biosynthetic reactions within various organisms. These are integral to both basal metabolism and the production of secondary metabolites that degrade exogenous chemical toxicity (Sun et al., 2017). CYP450s convert toxic herbicides into non-toxic compounds, playing a crucial role in the initial stages (as phase I) of herbicide metabolism (Siminszky, 2006). Specifically, CYP monooxygenases detoxify herbicides in crops like maize and sorghum (Barrett, 1995). Previous studies have reported herbicide-resistant plant *CYP450* genes. For instance, ALS gene target site mutations confer nicosulfuron resistance in foxtail grass (Huang et al., 2021).

Previous studies have shown that the sensitivity of maize to nicosulfuron is controlled by a single recessive gene (Kang, 1993; Williams et al., 2005). Specifically, a recessive *CYP450* gene was reported to regulate nicosulfuron resistance and metabolism in sweet maize (Pataky et al., 2009). *CYP81A9* was considered as a candidate gene for nicosulfuron sensitivity in maize through bulked segregant RNA-Seq (BSR-Seq) analysis (Liu et al., 2019). The objectives of this study were to (i) fine-map and clone maize nicosulfuron resistance gene (*CYP81A9*), (ii) search for different allelic variations of *CYP81A9* in susceptible and resistant materials, and (iii) functional analysis of *CYP81A9* using EMS premature termination mutant, knockout and overexpression transgenic plants.

## Materials and methods

### Plant materials

A total of 42 maize materials and two BC_1_ populations were planted at the Experimental Station of Shandong Agricultural University (36.19433°N, 117.11969°E). Field trials identified 22 nicosulfuron-resistant inbred lines (e.g., B73, 70384, K278, Zong31) and 20 susceptible inbred lines (e.g., JS188, J1399, 55347, 70379). The populations 70384/JS188//JS188 and 70973/70269//70269, comprising 1962 and 3000 individuals respectively, were used for mapping. **Supplementary Table 1** provides the nicosulfuron resistant and sensitive inbred varieties. **Supplementary Table 2** lists the primers used in the experiments. These include primers used for mapping.

The *CYP81A9* EMS translation termination mutant *ems4-06e182* was acquired from the maize EMS mutant bank at Qilu Normal University (http://maizeems.qlnu.edu.cn/).

### Phenotypic evaluation of resistance to nicosulfuron

The responses of maize plants to nicosulfuron were evaluated in the field. Each identification material was planted in at least one row (15-20 plants). In all trials, maize seeds were planted at a spacing of 10 cm between plants and 30 cm between rows. The experiments employed a randomized block design with three replicates. Nicosulfuron, sourced from Hebei Zhongbao Green Crop Technology Co., Ltd (China), was applied to maize at the four-leaf stage by a concentration of 80 mg/L. Meanwhile, overexpression transgenic plants were treated with 20-fold (1600 mg/L) nicosulfuron at the four-leaf stage. Injury symptoms were assessed 10 days post-application. Plants were categorized as tolerant, moderate, or susceptible to nicosulfuron based on mean injury leaves being < 5%, ≥ 5% and < 20%, or ≥20%, respectively (Williams et al., 2005).

### Fine mapping and allele testing

Sensitive plants were identified after nicosulfuron application in two BC_1_ populations. Genomic DNA (gDNA) from these susceptible plants was extracted for fine mapping. Based on previous results, genotypes were identified using molecular markers developed within the initial mapping interval (Hong et al., 2012). Recombinant plants were then sought to narrow the target gene location.

The F_1_ generation, derived from crossing JS188 and heterozygous (EMS4-06e182/-), was treated with 80 mg/L nicosulfuron.

### Haplotype analysis

The full-length *CYP81A9* gDNA was amplified using two primer pairs (**Supplementary Table 2**), and the PCR products were sequenced by Shanghai Biotech Biological Company (China). Sequencing quality was verified, and the amino acid sequence translated using Snap Gene and DNAMAN softwares.

### Allelic mutants generated by CRISPR-cas9 gene editing

The CRISPR-Cas9 vector for *CYP81A9* was constructed using a single-target editing strategy with a Plant Cas9/gRNA Plasmid Construction Kit (Beijing Vaisunlide Biotechnology Co, Catalog. No. VK005-02). The 20 bp target sequence for editing (5’-ACGACGTGAACTTCGCGAAC-3’) was located in the first exon of *CYP81A9*. The CRISPR-Cas9 work was performed by the Institute of Crop Science, Chinese Academy of Agricultural Sciences. Zong31, resistant to nicosulfuron, was used as the editing receptor. A marker (**Supplementary Table 2**) was designed to identify the editing effect of *CYP81A9*. Three pure transgenic lines were obtained after editing, and named CR-*CYP81A9-1*, CR-*CYP81A9-2*, and CR-*CYP81A9-3*.

### *CYP81A9* over-expressed in Zong31

The full-length *CYP81A9* cDNA was amplified and underwent homologous recombination with the P3301-35S-CDS vector, thereby generating the overexpression construct P3301-35S-CDS-*CYP81A9* (OE-*CYP81A9*). Transformation of maize Zong31 callus was mediated by *Agrobacterium tumefaciens*. A marker (**Supplementary Table 2**) was designed to characterize the transformation effect of the *CYP81A9* overexpression vector.

### Determination of ALS activity

The Plant ALS ELISA kit was used to measure the ALS activity in the roots, stems, and leaves of wild-type plants (Zong31) and CRISPR-Cas9-produced homozygous plants (CR*-CYP81A9*-1) treated with nicosulfuron for 24h. This kit employs a double antibody sandwich method to quantify ALS levels in samples. Microtiter plates were coated with purified ALS capture antibodies to prepare solid-phase antibodies. After washing, the complexes were stained with TMB substrate. TMB turns blue in the presence of the HRP enzyme and yellow under acidic conditions, with the color intensity directly proportional to the ALS content in the sample. The absorbance (OD) was measured at 450 nm using a Rayto RT-6100 microplate reader (Rayto Life and Analytical Sciences Co., Ltd., Shenzen, China). ALS activity in the samples was calculated using a standard curve.

### qRT- PCR analysis

Total RNA was isolated from the roots, stems, and leaves of nicosulfuron-resistant inbred line B73 and susceptible inbred line JS188 using an EasySpin Plus Plant RNA Kit (Aidlab, Beijing, China) at 10 time points following the 80 mg/L nicosulfuron application (0 h, 2 h, 4 h, 8 h, 12 h, 24 h, 48 h, 72 h, 96 h, and 192 h). High-quality first-strand cDNA was synthesized with the HiFiScript gDNA Removal cDNA Synthesis Kit (CW Biotech, Beijing, China). The maize actin gene served as an internal control. The primers sequences are listed in **Supplementary Table 2**.

### Statistical analysis

Data analysis was conducted using SPSS 22.0 software, with results from three independent biological replicates presented as mean ± SD (standard deviation). Group differences were assessed with a two-sided Student’s *t*-test, considering *p* < 0.05 as statistically significant. Additionally, Microsoft Excel 2016 was used for the Chi-square test (χ^2(0.05)^≤3.84).

## Results

### Fine mapping of *CYP81A9*

**Figure 1:**
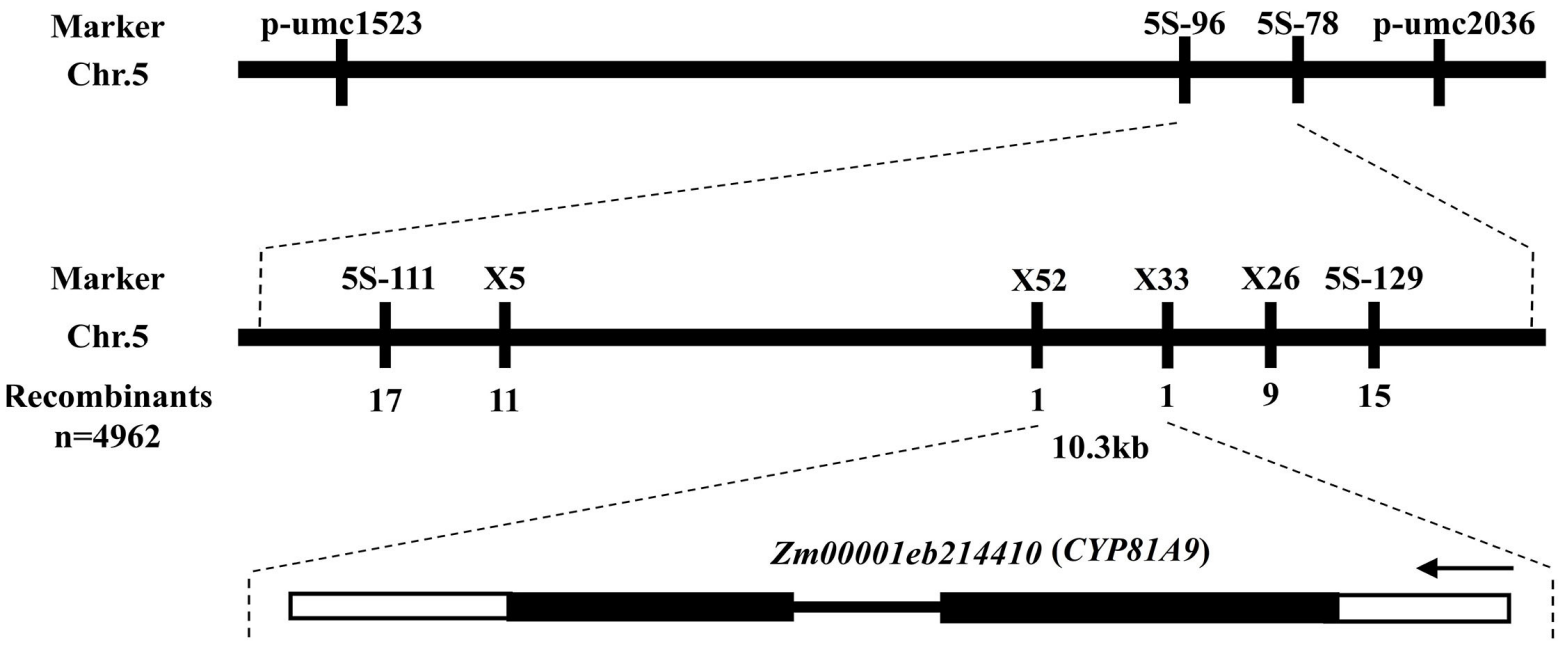
Mapping interval and the structure of candidate gene *CYP81A9*.

Nicosulfuron sensitivity is well known to be conditioned by a single recessive gene, thereby corroborating previous research (Kang, 1993; Nordby et al., 2008; Hong et al., 2012). Our earlier reports identified this gene on the short arm of chromosome 5, located between markers *5s-96* and *5s-78*, spanning a physical distance of 0.18 Mb (Hong et al., 2012). To refine this localization, we constructed two mapping populations comprising 4962 susceptible individuals. We designed six pairs of specific primers within the *5s-96* to *5s-78* region to co-segregate with the target gene. Ultimately, we narrowed the candidate gene to a 10.3 kb region between markers *X52* and *X33*. A search of the annotation database (http://www.maizesequence.org/index.html) revealed only one gene, *Zm00001eb214410* (*CYP81A9*), within this 10.3 kb region (**Figure 1**). Liu et al. (2019) also located the nicosulfuron sensitivity gene (*Nss*) in the 14.2 Mb region of the short arm of chromosome 5 using BSR-Seq technology, identifying *CYP81A9* as the candidate gene, which is consistent with our gene mapping results.

### Amino acid sequence alignment analysis of *CYP81A9*

**Figure 2:**
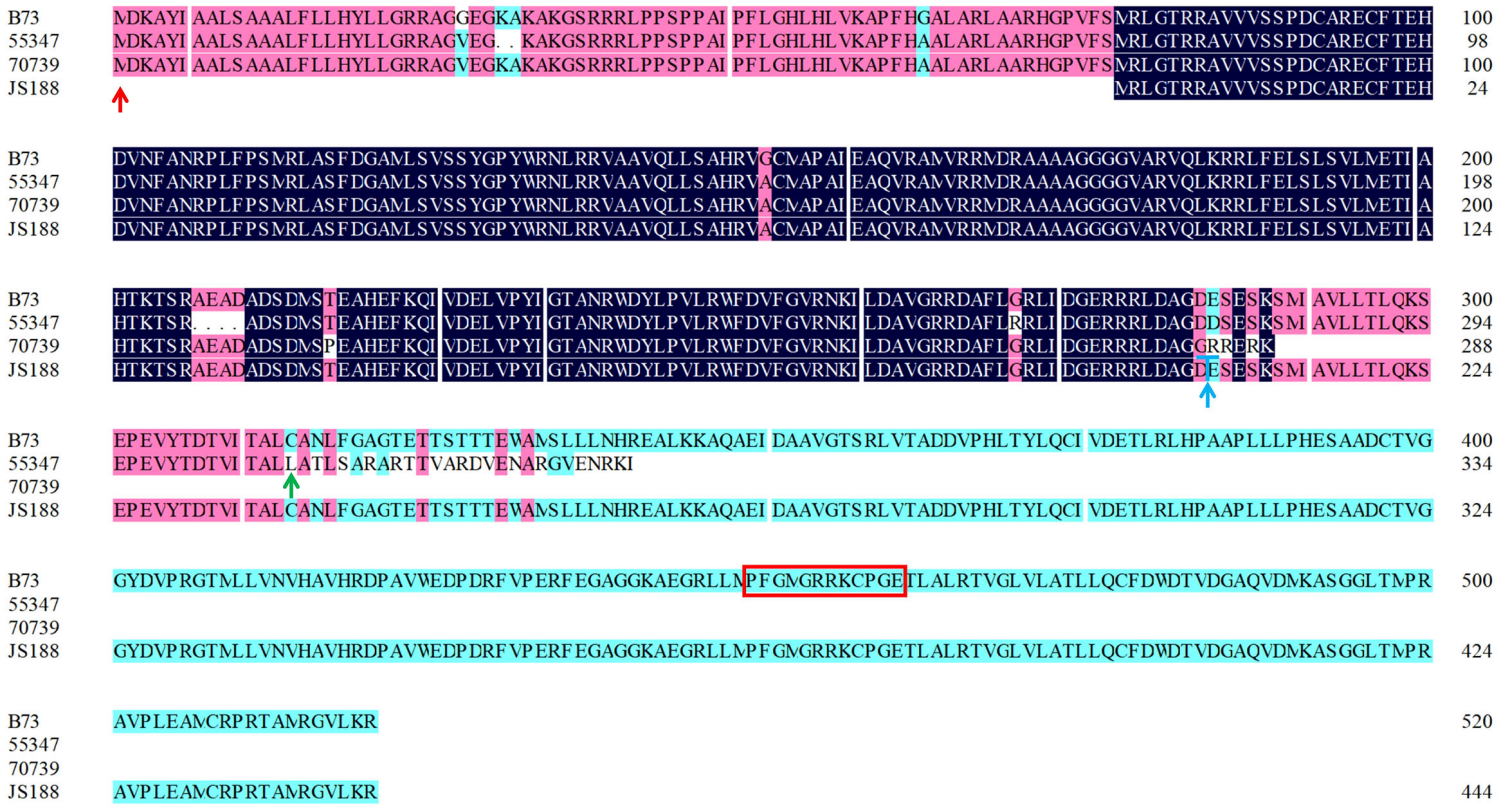
Amino acid alignment of CYP81A9_B73_, CYP81A9_55347_, CYP81A9_70379_, and CYP81A9_JS188_. The green, red and blue arrows indicate the amino acid mutation positions of CYP81A9_JS188_, CYP81A9_55347_, and CYP81A9_70379_, respectively, with the red boxes indicating the conserved sequences of the P450 family.

Subsequently, the gDNA sequences of 42 susceptible and resistant materials were sequenced and comparatively analyzed. The results revealed that there were some haplotypes including premature termination, frameshift stop and some point mutation in the susceptible material (**Supplementary Table 3**). Specifically, the haplotype *CYP81A9*_55347_ caused a disorder from amino acid position 307 to the end of the first exon and deletion of the second exon. The haplotype *CYP81A9*_70379_ premature termination led to a final deletion of 232 amino acids. Finally, haplotype *CYP81A9*_JS188_ had a 351 bp deletion in the 5’UTR and exon 1, resulting in the loss of the start codon ATG (**Figure 2**). These findings suggest that changes in the encoded amino acids alter the protein conformation. Consequently, the observed protein variations in the susceptible maize lead to the loss of CYP81A9 function, which consequently caused phytotoxicity.

### Functional analysis of *CYP81A9*

**Figure 3:**
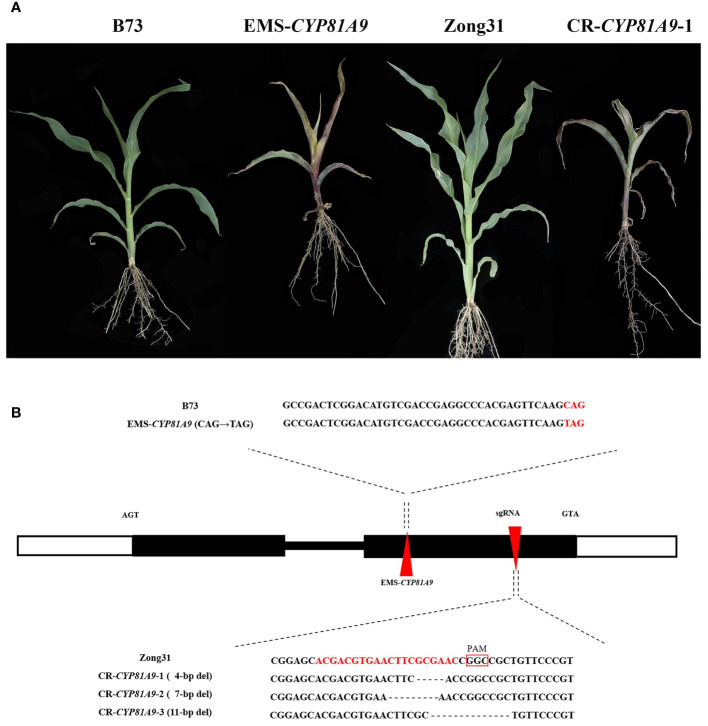
Validation of *CYP81A9*. **(A)** Performance of EMS mutant background material B73, EMS mutant, CRISPR/Cas9 mutant background material Zong31 and CR-CYP81A9-1 after 10 days treatment with nicosulfuron (80 mg/L); **(B)** Localization of EMS mutations and Cas9 knockouts and their base variant sequences. The red triangle shape is the mutation position of the mutant on *CYP81A9*.

To determine whether *CYP81A9* is a key gene in maize resistance to nicosulfuron, we obtained the *CYP81A9* EMS premature termination mutant (*EMS4-06e182*). Sequencing of this mutant revealed a C→T mutation at 670 bp in *CYP81A9*. Phenotypic test results showed that B73 maize (**Figure 3A**) grew normally, whereas *EMS4-06e182* homozygous mutants (**Figure 3A**) exhibited injury symptoms after 10 days of nicosulfuron (80 mg/L) treatment. Allelism tests between JS188 and *EMS4-06e182* showed a phenotypic segregation ratio of 1:1 (30/33, χ^2^ = 0.143, P(χ^2^)=0.9), with all F_1_ (JS188/*EMS4-06e182)* plants showing injury, confirming that *CYP81A9* is the target gene.

Using the CRISPR/Cas9 system, we designed a gene-specific sgRNA targeting *CYP81A9* and obtained three independent T_0_ knockout transgenic lines from the resistant line Zong31 (**Figure 3B**), named CR-*CYP81A9-1*, CR-*CYP81A9-2*, and CR-*CYP81A9-3*. Further nicosulfuron resistance evaluations were conducted on Zong31 (**Figure 3A**) and T_3_ homozygous mutants. After 10 days of nicosulfuron (80 mg/L) treatment, the CR-*CYP81A9* mutants lost resistance (**Figure 3A**), thereby confirming *CYP81A9* as the target gene. Li et al. (2013) demonstrated that maize plants with silenced *CYP81A9* were killed by conventional nicosulfuron dosages, further substantiating the critical role of *CYP81A9* in maize sensitivity to nicosulfuron.

**Figure 4:**
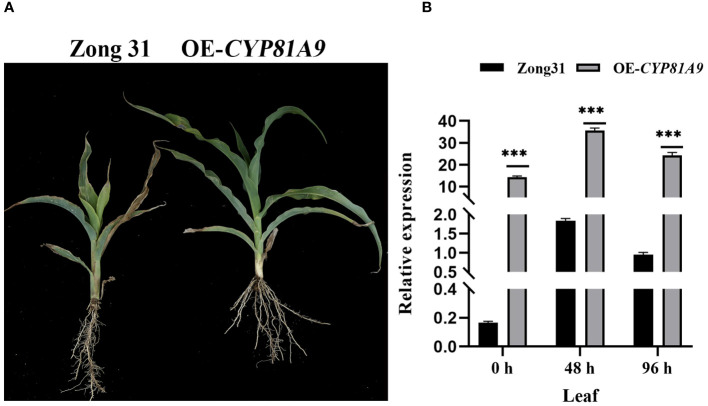
The nicosulfuron resistance detection and qRT-PCR analysis of transgenic material. **(A)** Performance of Zong31 and OE-*CYP81A9* 10 days after a 20× nicosulfuron (1600 mg/L) treatment. **(B)** The *CYP81A9* relative expression in leaves of Zong31 and OE-*CYP81A9* at different time points after nicosulfuron 20X (1600 mg/L) treatment. Values represent means of three repeats (***P < 0.001).

Furthermore, the *CYP81A9* overexpression vector was constructed and introduced into the resistant line Zong31 using Agrobacterium-mediated transformation. Phenotypic results demonstrated that both wild-type Zong31 and overexpression plants (OE-*CYP81A9*) grew normally after 80 mg/L nicosulfuron application (**Figure 3A**). However, at a nicosulfuron concentration 20 times the field application rate (80 mg/L), wild-type Zong31 were severely damaged, whereas overexpression plants remained resistant (**Figure 4A**). After 48 h of 1600 mg/L nicosulfuron treatment, both Zong31 and OE-*CYP81A9* showed significantly upregulated *CYP81A9* expression, with OE-*CYP81A9* showing the most significant increase (**Figure 4B**). Although the receptor material (Zong31) is a resistant inbred line, OE-*CYP81A9* produced higher expressed product and metabolized nicosulfuron more rapidly. Thus, overexpressing *CYP81A9* in Zong31 enhanced protection against high nicosulfuron concentrations. Therefore, these findings suggest that increased *CYP81A9* expression is linked to maize nicosulfuron resistance.

### The analysis of *CYP81A9* expression and ALS activity

**Figure 5:**
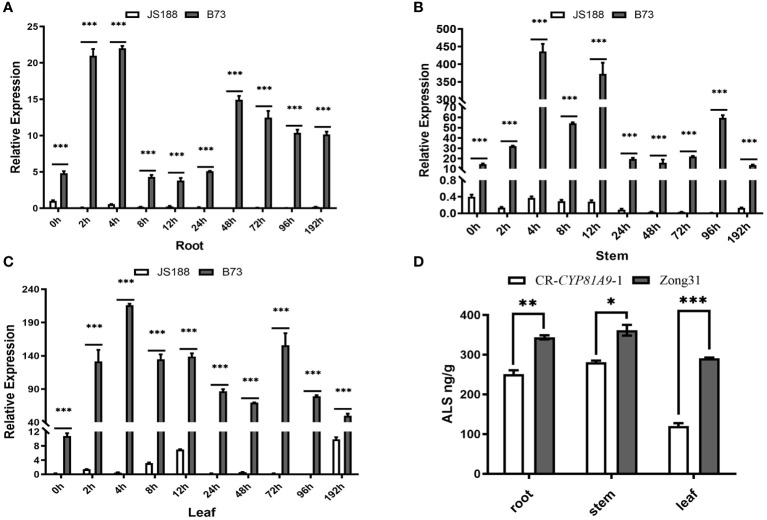
*CYP81A9* expression and plant ALS activity analysis **(A–C).** The relative expression levels of *CYP81A9* in roots, stems, and leaves of JS188 and B73 at different time points after nicosulfuron (80 mg/L) treatment. **(D)** Relative ALS content in CR-*CYP81A9* and Zong31. Values represent means of three repeats (*P < 0.05, **P < 0.01, ***P < 0.001).

To investigate *CYP81A9* expression in nicosulfuron-treated resistant material, the resistant inbred line B73 and the susceptible inbred line JS188 were sprayed at the 4-leaf stage. Samples from roots, stems, and leaves were collected at 10 time points post-spraying and analyzed using qRT- PCR. As shown in **Figures 5A–C**, *CYP81A9* expression levels in B73 were consistently higher than in JS188 across all 10 time points. In B73, *CYP81A9* expression peaked within 4 h in roots, stems, and leaves. These results indicate that *CYP81A9* is upregulated in resistant maize but minimally expressed in susceptible maize. Consequently, *CYP81A9* may be crucial in metabolizing and detoxifying nicosulfuron.

ALS activity was measured in the roots, stems, and leaves of Zong31 and CR-*CYP81A9-1* plants treated with nicosulfuron for 24 h. The results showed a significant decrease in ALS content in the roots, stems, and leaves of homozygous CR-*CYP81A9* plants compared to Zong31. The lowest ALS content was observed in the leaves (**Figure 5D**). As the leaf is the initial point of contact with the nicosulfuron, it likely experienced the most immediate and severe effects, resulting in a lower ALS content compared to other plant parts. Overall, we found that *CYP81A9* expression and ALS activity are significantly reduced in nicosulfuron-sensitive maize.

## Discussion

Weeds have always significantly limited maize yield, as they compete with maize plants for crucial resources such as nutrients, water, light, and space (Ferrero et al., 2017). As the maize planting area has increased, herbicides have become the primary method of weed control. Nicosulfuron is a widely used post-emergence herbicide for maize. However, over the past 20 years, extensive use of nicosulfuron has caused severe injury to certain maize cultivars like sweet corn, waxy corn, and popcorn (Green and Ulrich, 1993; Williams et al., 2005; Meyer et al., 2010; Wang et al., 2018). Studies have shown that the recommended nicosulfuron dosage significantly reduced (37.09%) the yield of the susceptible maize variety Ditian 8. In contrast, the resistant variety Nongda 108 maintained its yield, thereby demonstrating its resilience against the adverse nicosulfuron-induced effects (Lu et al., 2024). In this study, 20 susceptible inbred lines, including JS188, 70269, 55347, and 70973, were identified among 42 inbred lines, with most being waxy corn. Choe and Williams (2020) reported nicosulfuron injury in 106 sweet corn inbred lines with varying severity. Additionally, some common maize inbred lines, such as J1399 and 80162, also showed sensitivity to nicosulfuron. Among 223 public inbred lines, 25 were susceptible to nicosulfuron (Kang, 1993). Furthermore, prolonged use of a single herbicide can lead to significant weed resistance (Beckie and Tardif, 2012; Chuhan and Mahajan, 2014), complicating weed control with standard doses. Therefore, identifying nicosulfuron-resistant genes in maize is crucial. This study not only provides crucial insights into the mechanisms underlying herbicide resistance, but also it provides new genetic resources to cultivate herbicide-resistant maize varieties.

The nicosulfuron sensitivity is constrained by a single recessive gene, as indicated by previous studies (Kang, 1993; Nordby et al., 2008). Fine mapping results from this study suggest that *CYP81A9* is potentially the candidate gene responsible for nicosulfuron sensitivity in corn, encoding a cytochrome P450 monooxygenase. CYP450 is recognized for its role in xenobiotic metabolism, including herbicides, thereby protecting plants from phytotoxicity by modifying synthetic herbicides and insecticides and degrading harmful substances (Schuler and Werck-Reichhart, 2003; Nelson and Werck-Reichhart, 2011). In susceptible plants, sulfonylurea herbicides undergo alkyl or aryl hydroxylation reactions more frequently after the catalytic action of cytochrome P450 monooxygenase, accelerating herbicide metabolism and rapidly generating non-toxic, inactive compounds. The cytochrome P450 system plays a significant role in this process (Tan et al., 2006).

Li found that transgenic corn plants (R450-58) with silenced *CYP81A9* were susceptible to nicosulfuron at a 40 g/ha application dosage (Li et al., 2013). We demonstrated that *CR-CYP81A9* and EMS mutants lost nicosulfuron resistance at an 80 mg/L spray, while OE-*CYP81A9* maintained resistance at 20 times that concentration (1600 mg/L). *CYP81A9* showed upregulation in the resistant B73 inbred-line but minimal expression in the susceptible JS188 inbred-line. Notably, OE-*CYP81A9* expression in leaves was most significantly upregulated following a 1600 mg/L nicosulfuron spray, particularly after 48h, compared to Zong31 and *CR-CYP81A9-1*. The current study observed lower *CYP81A9* expression and ALS activity in susceptible maize material compared to Zong31. Furthermore, the tolerant maize hybrid DK689 rapidly metabolized 78–95% of nicosulfuron within the plant 72 h after spraying (Gallaher et al., 1999). The terminal oxygenase, isoflavone 2’-hydroxylase-like, encoded by *CYP81A9*, participates in isoflavone biosynthesis through a series of oxidation reactions in plants, animals, and microorganisms (Paul et al., 1994). This enzyme catalyzes the metabolism of nicosulfuron. A decrease in *CYP81A9* expression potentially impedes the rapid metabolism of nicosulfuron in susceptible maize, resulting in the inhibition of ALS enzyme activity and subsequent phytotoxicity. The expression of key genes, such as *CYP81A9*, is crucial for enhancing plant adaptability to nicosulfuron stress (Wang et al., 2021). In tolerant maize, the strong oxidase activity of *CYP81A9* facilitates the detoxification and metabolism of nicosulfuron, promptly degrading it into inactive substances. Consequently, tolerant maize sustains high ALS viability under nicosulfuron toxicity conditions (Nordby et al., 2008; Choe and Williams, 2020). These findings imply that *CYP81A9* represents a significant gene controlling maize tolerance to nicosulfuron.

Reports have indicated a 392-base pair insertion in the *CYP81A9* gene, causing maize sensitivity to nicosulfuron (Williams et al., 2005; Jerald et al., 2008). Our results showed that the nicosulfuron-sensitive inbred line 55347 contains a 392 bp insertion at 939 bp, consequently introducing a stop codon at 82 bp within the insertion region. This disruption generates a sequence alteration from amino acid position 307 to the end of the first exon and eliminates the entire second exon. The 392 bp insertion in nicosulfuron-sensitive maize produces a truncated protein, losing both substrate and heme binding sites (Williams et al., 2006). Additional *CYP81A9* haplotypes correlate with maize sensitivity to nicosulfuron. Analysis of susceptible inbred lines HB39 and HB41, relative to tolerant lines, uncovered three deletions, one insertion, and several point mutations (Liu et al., 2019), which align with observations in the 70269 line from this study. Similarly, this study identified a TGGA insertion at 846 bp in the nicosulfuron-sensitive inbred line 70379, triggering premature termination and a 232 amino acid deletion. Sequence alignments revealed that base mutations conferred nicosulfuron sensitivity in maize. These mutations signified that the *CYP81A9* structure and expression changes were associated with maize’s resistance to nicosulfuron. We posited that these alterations led to changes in the conformation of the synthesized protein due to amino acid modifications. This protein change resulted in the loss of *CYP81A9* gene function in susceptible maize, culminating in phytotoxicity. In contrast, resistant maize varieties maintain normal *CYP81A9* function, enabling nicosulfuron degradation and preventing drug-induced damage. The CYP81A9 enzyme metabolizes nicosulfuron in sweet corn, with varying amino acid changes in the *CYP81A9* sequence corresponding to differing levels of nicosulfuron injury (Choe, 2020). Therefore, a thorough examination of *CYP81A9* alleles and the selection of tolerant alleles can enhance nicosulfuron tolerance in maize. This approach would facilitate the cultivation of maize varieties with greater resilience against nicosulfuron applications, thereby minimizing the risk of phytotoxicity and maximizing crop yields.

## Conclusions

In summary, this study identified *CYP81A9* via map-based cloning and functional validation as the key gene regulating nicosulfuron resistance in maize. Moreover, we also discovered different haplotypes of *CYP81A9*. Overall, our study provides a solid foundation for further exploration of the underlying nicosulfuron resistance mechanisms in maize.

## Data availability statement

The raw data supporting the conclusions of this article will be made available by the authors, without undue reservation.

## Author contributions

YZ: Writing – review & editing, Writing – original draft. QZ: Writing – review & editing, Writing – original draft. QL: Writing – review & editing, Writing – original draft. YZ: Writing – review & editing, Writing – original draft. WX: Writing – review & editing, Writing – original draft. CH: Writing – review & editing, Writing – original draft. CX: Writing – review & editing, Writing – original draft. XQ: Writing – review & editing, Writing – original draft. BL: Writing – review & editing, Writing – original draft.
